# Evaluating patient-reported outcome measures (PROMs) for clinical trials and clinical practice in adult patients with uveitis or scleritis: a systematic review

**DOI:** 10.1186/s12348-022-00304-3

**Published:** 2022-09-05

**Authors:** Charles O’Donovan, Jesse Panthagani, Olalekan Lee Aiyegbusi, Xiaoxuan Liu, Susan Bayliss, Melanie Calvert, Konrad Pesudovs, Alastair Denniston, David Moore, Tasanee Braithwaite

**Affiliations:** 1 School of Immunology and Microbiology, King’s College London, London, England; 2 University Hospitals Birmingham, Birmingham, England; 3 Centre for Patient Reported Outcomes Research (CPROR), Institute of Applied Health Research, Birmingham Health Partners for Regulatory Science and Innovation, and NIHR, Birmingham Biomedical Research Centre, University of Birmingham, Birmingham, B15 2TT UK; 4 Institute of Inflammation and Ageing, University of Birmingham, University Hospitals Birmingham, Birmingham, England; 5 Health Data Research UK, London, England; 6 Institute of Applied Health Research, University of Birmingham, Birmingham, England; 7 Centre for Patient Reported Outcomes Research (CPROR), Institute of Applied Health Research, Birmingham Health Partners for Regulatory Science and Innovation, NIHR, Birmingham Biomedical Research Centre, NIHR Surgical Reconstruction and Microbiology Centre and NIHR Applied Research Collaboration West Midlands, University of Birmingham, Birmingham, B15 2TT UK; 8 University of New South Wales, Kensington, Australia; 9 Vision and Eye Research Institute, Anglia Ruskin University, Cambridge, UK; 10 The Medical Eye Unit, Guy’s and St Thomas’ Hospital NHS Foundation Trust, London, England

## Abstract

Patient reported outcome measures (PROMs) capture impact of disease and treatment on quality of life, and have an emerging role in clinical trial outcome measurement. This study included a systematic review and quality appraisal of PROMs developed or validated for use in adults with uveitis or scleritis. We searched MEDLINE, EMBASE, PsycINFO, CINAHL and grey literature sources, to 5 November 2021. We used established quality criteria to grade each PROM instrument in multiple domains from A (high quality) to C (low quality), and assessed content development, validity, reliability and responsiveness. For instruments developed using classic test theory-based psychometric approaches, we assessed acceptability, item targeting and internal consistency. For instruments developed using Item Response Theory (IRT) (e.g. Rasch analysis), we assessed response categories, dimensionality, measurement precision, item fit statistics, differential item functioning and targeting. We identified and appraised four instruments applicable to certain uveitis types, but none for scleritis. Specifically, the National Eye Institute Visual Function Questionnaire-25 (NEI-VFQ), a 3-part PROM for Birdshot retinochoroiditis (Birdshot Disease & Medication Symptoms Questionnaire [BD&MSQ], the quality of life (QoL) impact of Birdshot Chorioretinopathy [QoL BCR], and the QoL impact of BCR medication [QoL Meds], the Kings Sarcoidosis Questionnaire (KSQ), and a PROM for cytomegalovirus retinitis. These instruments had limited coverage for these heterogeneous conditions, with a focus on very rare subtypes. Psychometric appraisal revealed considerable variability between instruments, limited content development, and only one developed using Item Response Theory. In conclusion, there are few validated PROMs for patients with uveitis and none for scleritis, and existing instruments have suboptimal psychometric performance. We articulate why we do not recommend their inclusion as clinical trial outcome measures for drug licensing purposes, and highlight an unmet need for PROMs applicable to uveitis and scleritis.

**Supplementary Information:**

The online version contains supplementary material available at 10.1186/s12348-022-00304-3.

## Introduction

Finding effective treatments for rare diseases, and specifically, for uveitis and scleritis has been identified as a research priority by stakeholders internationally [1, 2].The acutely sight-threatening nature of these ocular inflammatory disorders, and their frequently chronic or relapsing course, means that systemic therapies are often required. Many patients have associated immune-mediated inflammatory disease (IMID), of infectious, or non-infectious (autoimmune or autoinflammatory) aetiology [3]. There have been significant advances in therapeutic options in recent years. Biologic therapies are complementing traditional use of corticosteroids and second-line immunosuppressive therapy for some indications, including non-infectious posterior, intermediate and panuveitis. Ongoing development or repurposing of biologic therapies, targeting underlying loss of immune tolerance or early instigators of the inflammatory cascade, is likely to transform management [4]. Treatments, whether initiated for eye disease, or an associated IMID, must be assessed for their multisystem benefits and side effects. There is clear need to consider patients holistically and to take a multidisciplinary, multispecialty approach to care and outcome measurement that extends beyond traditional visual function and ocular imaging measures.

There has been growing focus on patient-centred definitions of efficacy, and better integration of the patient voice into research priority setting, outcomes design, and routine clinical practice in ophthalmology [5–7]. A patient reported outcome measure (PROM) facilitates quantitative capture of the subjectively experienced impacts of disease and its treatment (See Table 1 Glossary). For PROMs to be useful and acceptable, especially for drug marketing authorisation [7, 8], they need to be targeted to the constructs of interest, possess sound psychometric performance properties (e.g. as assessed using Item Response Theory models), and be valid, reliable, responsive and acceptable to users [9].Well-designed PROMs yield an interval-scaled measure for each quality of life domain measured, which is amenable to quantitative statistical analysis, and thus of tremendous value to clinicians and researchers [10].

**Table 1:** Glossary of key terms

**Patient reported outcome measure (PROM)**	PROMs are sets of questions or ‘items’ which form an ‘instrument’ used to quantify the subjective impacts of disease or its treatment. They can be broadly split into generic health, vision-specific, or disease-specific measures. Generic health measures usefully support comparison of the health status of different disease groups, whilst vision and disease-specific PROMs, including instruments focused on signs or symptoms, offer more sensitive measurement of change in health status
**Quality of life (QoL)**	QoL is a multidimensional construct, with domains potentially including symptoms relating to vision function, eye disease, or other aspects of health and organ function, impacts of disease and treatment on aspects of daily functioning, including daily activities, mental, social, emotional and economic functioning
**Classic test theory**	Classic test theory (CTT), also known as true score theory, is a quantitative approach to test the reliability and validity of a scale based on items. It considers the relationship between the expected score (or ‘true’ score) and observed score on any given measurement. The true score is one that would be obtained if there were no errors in measurement. It assumes that random errors (i.e., the difference between a true score and a set of observed scores on the same individual) are normally distributed and item responses are coded so that higher responses reflect more of the concept
**Item response theory (IRT)**	Item Response Theory (IRT) refers to psychometric statistical model that attempts to map data observed on participants to latent traits assumed to be causing the observations, in order to explain as much of the observed variance as possible. IRT assumes that the latent construct and items of a measure are organised in an unobservable continuum and its main purpose focuses on establishing the individual’s position on that continuum. As in CTT, IRT requires each item be distinct from the others, yet similar and consistent with them in reflecting all important respects of the underlying construct
**Rasch model**	The Rasch Model measures latent traits (like difficulty with daily vision-related tasks) and provides an internally valid measure by allowing non-linear raw data to be converted to a linear scale, which then can be evaluated through the use of parametric statistical tests. It assumes that the probability of a given person/item interaction is governed by the difficulty of the item and the ability of the person, that are determined by the item locations on the presumed latent variable along with the rating scale structure
**Principal Component Analysis (PCA)**	Principal Component Analysis (PCA) is a dimension-reducing tool that replaces the variables in a data set by a smaller number of derived variables

There is a pressing need for robust PROMs in inflammatory eye disease [6]. Denniston et al. reviewed uveitis clinical trials and reported that none included a PROM as the primary outcome measure [11]. More recent uveitis trials (e.g. SYCAMORE, VISUAL I and VISUAL II, MUST) include a variety of generic and vision-specific PROMs [12–16]. This timely systematic review aimed to identify and psychometrically evaluate the quality of all PROMs developed or validated in adults with scleritis or uveitis.

## Methods

The methodology followed our published PROSPERO protocol (CRD42019151652) [17]. The systematic review is reported in line with PRISMA guidance [17, 18].

### Searches

We systematically searched the following electronic databases on 5 November 2021: MEDLINE (Ovid), EMBASE(Ovid), PsycINFO (Ovid) and CINAHL Plus (EBSCO). The search strategy combined index and free text terms for the clinical entities, and terms relating to quality of life, health status indicators or patient-reported outcomes, with no restrictions on the language or year of publication (See supplement). The MEDLINE search strategy was adapted for use on all databases. We screened references of included studies, to identify any additional instruments. Where multiple studies referenced the same PROM, we searched citations to obtain the study reporting the original PROM’s development and any subsequent revisions and reports relating to instrument quality appraisal or validation.

### Study selection

We included studies reporting content identification, development, psychometric assessment, or validation of PROMs to assess the impact of uveitis or scleritis alone, or in combination, in adult patients. We included broad search terms for patient-reported outcomes and ‘quality of life’, considering ‘quality of life’ as an umbrella term including multiple domains (see Table 1). We sought studies that used valid disease-relevant content development methods such as structured/semi-structured interviews, focus groups and/or literature reviews, but did not exclude validation studies with weaker content development (e.g. expert opinion). We excluded editorials, reviews, conference abstracts and studies reporting instruments developed solely for use in children. We excluded studies reporting the use, but not the development of a PROM.

### Main outcomes

For each included study, we extracted study characteristics (publication year, citation, country/region, sample size) and characteristics of patients on whom the instrument was developed / assessed / validated. This included disease type(s) and subtypes, age, sex, ethnicity, and, if reported, the proportion of patients on systemic antimicrobial or anti-inflammatory therapy. We extracted the name of the PROM, the QoL domains covered, the number of items in each domain, and any subtypes of uveitis or scleritis covered by the PROM.

### Data extraction, synthesis and analysis

Search results were uploaded to Endnote X9 (Clarivate Analytics). All titles and abstracts were screened by two independent reviewers (TB and XL/CO), to remove irrelevant articles. Full text articles were obtained for studies that potentially met eligibility criteria. Abstracts that did not provide the reviewers with sufficient information to make a decision were taken forward for full-text screening, to minimise the risk of missing a potentially relevant article. At any stage, if the reviewers were unable to reach consensus, an additional reviewer was consulted (KP). Two reviewers (TB and OLA/JP/CO) independently extracted data from studies meeting the inclusion criteria, using a standardised form.

### PROM quality assessment

Two reviewers (TB and OLA/CO), with adjudication by a third (KP), considered the overall extent to which the instrument’s items were relevant to uveitis or scleritis, based on the patient samples used for item identification and development, and for instrument validation. We graded this as very relevant, somewhat relevant, or not very relevant.

We assessed the quality of each identified PROM using established quality criteria (see Supplementary Table 1 definitions), adapted from the US Food and Drug Administration framework and guidelines [19], and COSMIN Standards for the selection of health status Measurement Instruments [20, 21], grading each of multiple domains from A (high quality) to C (low quality) [22]. The framework has been used previously to appraise the quality of PROMs in ophthalmology [9, 23], including retinal disease [23], cataract [24], refractive surgery [25], refractive error [26], amblyopia and strabismus [27] and keratoconus [28]. We reviewed instrument content development, and appraised item identification and item selection. For item identification we assigned a grade ‘A’ for, “comprehensive consultation with patients,” if a sufficient number (i.e. more than 30) of relevant patients were included to achieve content saturation [29]. For item selection, we assigned a grade ‘A’, based on the COSMIN guidelines, if the pilot instrument contained more than 7 times the number of patients than items in the instrument (or in the case of multidimensional instrument, 7 times the number of items in the largest domain representing a unidimensional construct); if the patient sample was fewer than 5 times the number of items we graded this domain ‘inadequate’ (grade ‘C’) [30].

For instruments developed using classic test theory-based psychometric approaches, we assessed acceptability, item targeting and internal consistency, but we highlighted as a limitation that more modern psychometric approaches had not been considered (highlighting Table 2 cells in red for ‘not done’). For instruments developed using the more rigorous Item Response Theory (IRT) (e.g. Rasch analysis) approaches, we assessed response categories, dimensionality, measurement precision, item fit statistics, differential item functioning and targeting [10].

**Table 2:** Characteristics of Included Studies

First authorYear	Instrument and year of development	Domains/scales of QoL	Items	Uveitis relevant items, n	Country	Patients, N	Patient characteristics	Completion time
Uveitis								
Barry 2014 [25]	3 PROMS: BD&MSQ Qol BCR Qol Meds	BD&MSQ 8 (lights, vision, skin, body pain, breathless, mood, sleep, hair loss) QoL BCR 4 (mood/ relationship, daily activities, family relations, feeling unwell) QoL Meds 3 (mood/ relationships, vision, low mood)	BD&MSQ 21 QoL BCR: 20 QoL Meds: 12	BD&MSQ:7 QoL BCR:3 QoL Meds: 4	UK, national patient group	2 patients for initial content; 8 for instrument development; 150 for validation plus 33 healthy controls	Birdshot chorioretinopathy patients (*n* = 152) Mean age 53.1 (SD 9.6) years, 73.0% female	Not reported
Wu 1996 [26]	CMV Retinitis (no formal instrument name), 1992	Visual symptoms (5 items); visual function (7 items); global vision (2 items), impact of treatment (4 items)	18	14	USA, 7 sites	18 to develop item content; 26 to develop and validate instrument	CMV retinitis patients with AIDS (*n* = 26). Mean age 38.5 (SD 5.9) years, 96% male, 88% White	5 min
Martin 2001 [27]	CMV Retinitis (no formal instrument name, Wu 1996) VALIDATION STUDY	This study included 4 scales with 16 items from Wu et al. 1996: Visual symptoms (5 items), visual function (7 items), global vision (2 items); impact of treatment (2 items)	16	14	USA, 12 sites, 1992–1995	279 patients recruited in the CRRT trial	CMV retinitis patients (*n* = 279). Mean age 38.6 years, 91.8% male, 60.6% White	Not reported
Mangione 2001 [29]	NEI VFQ-25	9 subscales: General health (1 item), general vision (1 item), near vision (3 items), distance vision (3 items), ocular pain (2 items), colour vision (1 item), peripheral vision (1 item), driving (3 items); and VISION-SPECIFIC social functioning (2 items), mental health (4 items), dependency (3 items), and role limitations (2 items)	26	25	USA	Data from *n* = 262 pilot study and *n* = 597 field test who completed an earlier 51-item instrument, for validation	*N* = 262 of which *n* = 21 CMV retinitis. Mean age 61 years, 54% female, 81% White *N* = 597 of which *n* = 37 CMV retinitis. Mean age 64 years, 59% female, 63% White	Aimed to be 5 min, but not reported
Naik 2013 [30, 31] Normative comparison [32]	NEI-VFQ-25 for uveitis VALIDATION STUDY	General health 1 plus 25-items as above (Mangione 2001)	26	25	18 countries, 46 sites	*N* = 224 HURON RCT patients compared to NEI VFQ data from *n* = 122 [29]	Patients (*n* = 224) including 188 with intermediate, and 44 with posterior uveitis. Mean age 44.6 (SD 14.3) years, 63.4% female, 60.3% White	Not reported
Patel [28]	KSQ	5 modules: general health status, lung, skin, eye and medication	29	7 in eye module, with 10 General Health Status forming unidimensional set	UK	Content: 23 (including 7 with ocular sarcoidosis) Development and validation: 207 patients	100% sarcoidosis, *n* = 45/207 with ocular involvement	Mean 10 (SD 8) minutes
Scleritis								
No studies								

In both study types, we assessed validity (concurrent, convergent, discriminant and known group validity), reliability (test–retest) and responsiveness (See Supplementary Table 1 for definitions). Where the patient sample used to validate the instrument was not independent from the sample used to develop it (across one or more published papers) we highlighted this as a limitation of the instrument.

### Analysis of subgroups or subsets

We present the instruments developed for uveitis or scleritis, and any disease-specific causes separately.

## Results

**Fig. 1:**
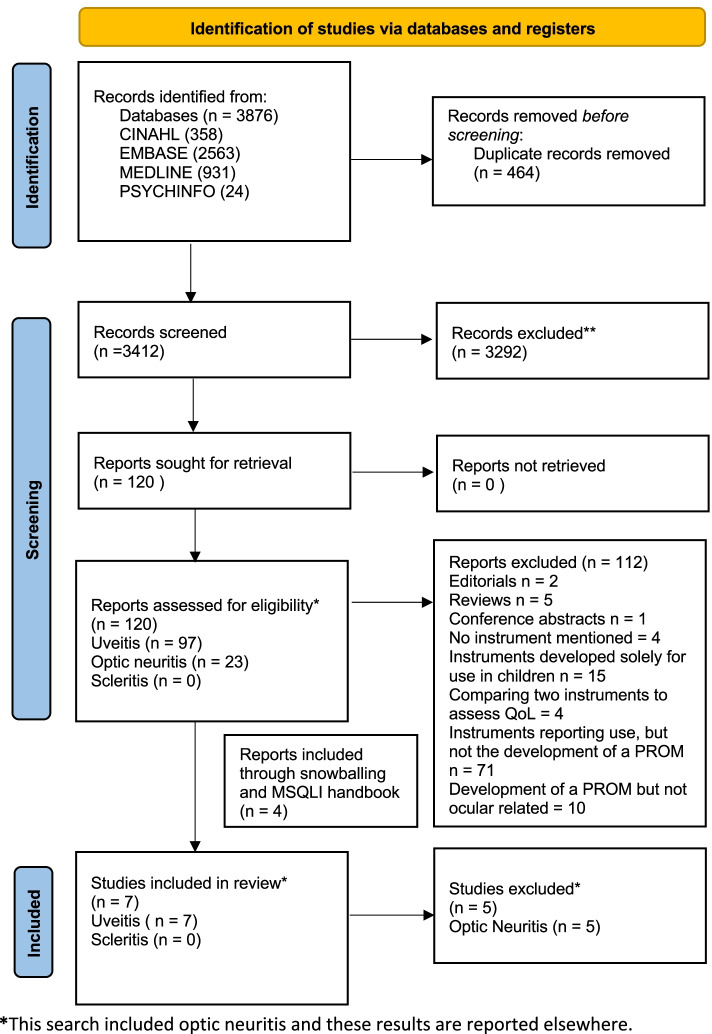
PRISMA flow diagram

The systematic search of bibliographic databases and cited references identified 3876 hits, reducing to 3412 after removal of duplicates. The study selection process is presented in Fig. 1. In total, for uveitis, we identified seven studies reporting four instruments. Specifically, an instrument developed and validated for Birdshot retinochoroiditis, an instrument developed and validated for cytomegalovirus retinitis associated with HIV infection, an instrument developed and validated for sarcoidosis (including ocular sarcoidosis, a cause of uveitis), and an instrument validated for non-infectious posterior and intermediate uveitis (using a previously developed vision-specific instrument).

No studies reported instruments for scleritis [31–37].

Table 2 summarises the characteristics of the included studies. Table 2 summarises the findings comparing the psychometric quality appraisal of included uveitis studies, against our predefined criteria (eTable 1). A justification of each grading assigned is available (Supplementary Table 2).

**Table 3:**
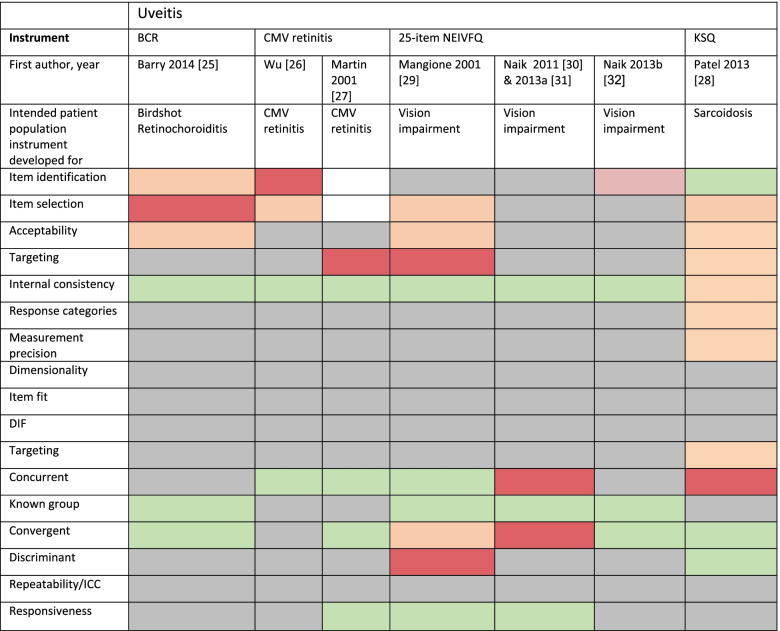
Psychometric quality appraisal of included studies

Green A, Orange B, Red C Grey: Not reported or not done

### UVEITIS PROMs

#### NEI VFQ-25 and its validity in uveitis

The original National Eye Institute Visual Function Questionnaire-25 (NEI VFQ-25) was developed between 1994 and 1998 for English-speaking adults aged >  = 21 years with vision impairment from age-related macular degeneration, cataract, diabetic neuropathy, glaucoma or cytomegalovirus retinitis (a type of infectious pan or posterior uveitis), following initial content development with multi-condition focus groups [38, 39]. A total of 262 patients were recruited from 5 academic centres, then a further 597 people were recruited in 1996 from multi-condition focus groups (only 5% *n*= 37/597 had cytomegalovirus retinitis). The original 51-item instrument was developed from a 96-item pilot instrument, and took 15 min to administer. The shorter 25-item NEI VFQ-25 was developed in 2001 [35]. This included 11 vision-related subscales (general vision, near vision, distance vision, driving, peripheral vision, colour vision, ocular pain, vision-specific role difficulties, vision-specific dependency, vision-specific social functioning, and vision-specific mental health) and one general health item. Each subscale was scored so that 0 represented the lowest and 100 the best possible score.

We graded the original NEI VFQ-25 with ‘A’ for item selection in its intended purpose, as an eye disease-generic vision-specific tool. However, as a uveitis-applicable tool we graded the instrument ‘C’. It is critical to note that the majority of focus group participants had other ophthalmic conditions, and those with uveitis had a rare and specific type of uveitis (CMV retinitis associated with HIV infection). Based on the item development process we would anticipate poor generalisability to uveitis in general, given few ‘appropriate patients’ for uveitis. We scored NEI VFQ-25 ‘A’ for internal consistency based on classic test theory, but ‘B’ for acceptability and ‘C’ for targeting. All 4 types of validity were assessed, but only concurrent and known group validity were graded ‘A’ in this tool’s capacity as an eye disease-generic vision-specific instrument, with convergent validity graded ‘B’ and discriminant validity graded ‘C’. However, the validation was not specific to uveitis.

We identified three papers by Naik et al. which sought to validate use of the NEI VFQ-25 for non-infectious intermediate and posterior uveitis. Two reports from one study used secondary analysis of data (*n*= 224) from the HURON trial, a multicentre Phase 3 randomised controlled trial (RCT) assessing the efficacy and safety of dexamethasone intravitreal implant (Ozurdex) compared to sham [37]. This validation study did not include any item generation, perpetuating the limitations of the earlier instrument. We graded the instrument ‘A’ for internal consistency based on CTT approaches, but targeting and acceptability were not reported. Known group validity was graded ‘A’ but convergent and concurrent validity ‘C’, with no assessment of discriminant validity. Based on an additional study report with comparison to normative data from a normal reference population (*n*= 122) we revised the grading of convergent validity to ‘A’ [36].

#### Birdshot retinochoroiditis PROMs

We identified one study by Barry et al. reporting a PROM for Birdshot retinochoroiditis including three domains; the Birdshot Disease & Medication Symptoms Questionnaire (BD&MSQ, 43 items in pilot, 21 in final); the QoL impact of Birdshot Chorioretinopathy (BCR) disease (QoL BCR, 25 items in pilot, 20 in finals); and the QoL impact of BCR medication (QoL Meds, 25 items in pilot, 12 in final) [31]. Content development was limited to an expert panel of two patients, one ophthalmologist and a psychologist, and did not explicitly reference a literature review (although it is highly unlikely there was any relevant prior literature for this rare disease). Instrument development used factor analysis to identify subscales from the responses of eight patient volunteers and one normal control, before validation in a larger sample of 150 patient volunteers recruited via the UK’s national patient support group. However, the factor analysis used for item selection was arguably invalid, including an insufficient number of responses from only 8 patients (more questions than people) so we downgraded this to C. We graded the internal consistency ‘A’, but judged approaches to item identification and assessment of acceptability to be Grade B. The study assessed 2 out of 4 domains of validity well (Grade A), but did not assess temporal responsiveness/reliability.

#### CMV retinitis PROM

The 18-item instrument for patients with cytomegalovirus (CMV) retinitis associated with acquired immunodeficiency syndrome (AIDS) was developed in 1992 for the ‘Ocular Complications of AIDS Foscarnet-Ganciclovir CMV Retinitis’ Trial [32]. The key limitations of this instrument were that, whilst some qualitative research was undertaken, most of the 44 items in the pilot instrument were repurposed from other non-relevant studies including the Visual Function-14 (VF-14) instrument, developed to assess visual function and symptoms in patients with cataracts [40]; the Medical Outcomes Study Short Form [41]; and the SF-36 [42]., and Classic Test Theory approaches were used to develop the final 18-item instrument. Martin et al. subsequently validated the CMV retinitis-specific QoL instrument [32], in an independent sample of 279 patients included in the CRRT multicentre, randomized controlled trial of intravenous foscarnet, intravenous ganciclovir and combination treatment for relapsed CMV retinitis. We again graded the instrument validation data with A for internal consistency using the CTT approach, and A for concurrent validity, with this study adding convergent validity (Grade A), and responsiveness (Grade A), but revealing poor targeting (Grade C).

#### King’s Sarcoidosis questionnaire

The 29-item King’s Sarcoidosis Questionnaire (KSQ) developed in 2011 assessed impact of sarcoidosis and its treatment on ocular symptoms (7 items) and general health state (10 items), amongst others, in the past 2 weeks. (34) This was the only instrument we identified which used IRT (the Rasch model) for item selection and instrument development, and which confirmed good score repeatability 2 weeks later. We assessed the ocular item set specifically, and felt this performed well in most aspects of the quality appraisal (Table 3 and eTable 2). Key limitations were that: content was developed from interviews with just 7 ocular sarcoidosis patients (with uveitis subtype unspecified), which we felt was unlikely to be sufficient to achieve content saturation; the patient sample (*n* = 207) was small for the initial 65 items under investigation; and the validation study of the final 29-item instrument did not use an independent sample.

## Discussion

This systematic review identified a paucity of disease-specific PROMs for use in uveitis (*n* = 4) and no PROMs for scleritis. There was very limited coverage of relevant diseases to ocular inflammatory disease phenotypes, with focus on cytomegalovirus retinitis associated with HIV infection (which was an important concern at the time, but is now a rare presentation thanks to anti-retroviral therapy), sarcoidosis, and Birdshot retinochoroiditis. No PROM covered the most frequent manifestation of inflammatory eye disease, namely, anterior uveitis. This aligned with our expectation, given the lack of inclusion of disease-specific PROMs in recent and currently ongoing RCTs.

Our quality appraisal revealed numerous limitations of the available instruments, with few instruments scoring a good grade ‘A’ in multiple domains. In contrast to other areas of ophthalmic PROM development, contemporary psychometric approaches incorporating item response theory have seldom been used in uveitis PROMs; the notable exception was the KSQ which was developed by respiratory physicians, for use aligned to the systemic condition rather than ocular sarcoidosis per se. Petrillo and colleagues argue that there are multiple issues with using classic test theory for psychometric evaluations [43]. Specifically, analysis is not based on interval-level measurement but on counts (summary scores of items), findings are dependent on the scale and sample, missing data cannot be handled easily, and the standard error of measurement around individual patient scores are assumed to have a constant value. Contemporary psychometric tools, such as Rasch Measurement Theory, permit a more robust approach to examination of validity and interpretability. This is recommended, especially if a PROM is being developed for the high-stakes situation of a pharmaceutical labelling claim. Many of these studies were developed and validated many years before the widespread application of COSMIN guidelines and IRT-based quality appraisal tools, and so it is not surprising that these older studies have been assessed to have suboptimal quality by contemporary standards. It is worth noting that not all the quality assessment criteria in eTable 1 are of equal value and importance. The possession of interval scaling and Rasch validity (especially precision and unidimensionality) is much more important than assessments of validity, reliability, or acceptability. For without interval scaling, the PROM is effectively not quantitative and therefore it will not find impactful applications.

Consideration of the NEI VFQ-25 helps to illustrate these points. The NEI VFQ-25 was not developed for uveitis specifically (5% of people providing content input had intermediate or posterior uveitis resulting from CMV infection in HIV) [44]. It has been frequently included as a secondary outcome measure in clinical trials in ophthalmology, and in uveitis [6]. However, multiple studies have psychometrically evaluated the NEI VFQ-25 in patients with different ocular conditions and the general population, and have identified major shortcomings with respect to reliability, validity and dimensional structure [45–49]. Exploring data from 2487 patients with retinal disease, Petrillo et al. reported that the NEI-VFQ-25 contained disordered response thresholds (15/25 items) and mis-fitting items (8/25 items) [50]. The psychometric performance has been similarly critiqued in low vision and cataract populations, with studies identifying only two unidimensional scales individually fitting the Rasch model [47, 48].A Rasch re-engineered NEI VFQ with two domains and fewer items has been developed [48, 49], but has not been validated in uveitis or scleritis. The FDA have noted the lack of validated PROMs in ophthalmology, and indicated that none of those used in trials to date would be considered acceptable for drug licensing purposes [51].

A further general theme emerging from this review was the exceedingly small number of patients interviewed to obtain item content for inclusion, ranging from 2 (Birdshot retinochoroiditis PROM) to 37 in NEI-VFQ [31, 52].This likely reflects the resources and expertise needed to conduct this form of qualitative research. Typically, the COSMIN guidelines suggest that more than 100 relevant patients are needed to develop ‘very good’ content for a structurally valid PROM (at least 7 times the number of items); whereas if the patient sample is fewer than 5 times the number of items in the instrument to be validated, this is ‘inadequate’ [30]. A key unanswered question is whether PROMs, developed without extensive content identification in far larger numbers of patients, have adequate external generalisability to other settings (different countries, demographics, disease subtypes and treatments). Furthermore, we note that the quality appraisal criteria (Supplementary Table 1) do not account for whether the patients included in content development were relevant to the outcome of interest for which their quality is being appraised.

Also evident was a historic desire for short instruments with completion times around five minutes to minimise participant burden, in the context of clinical trial examination protocols. Quality appraisal indicates that this focus on speed may have come at the cost of psychometric instrument performance. Evidence suggests there are at least 10 domains of quality of life relevant to people with ophthalmic diseases, extending beyond, but including symptoms of disease (see Table 1). Each domain of interest needs to be measured with a sufficient number of items, spread out on an interval scale, to yield a precise measure for that domain. This is impossible when only one item is included per scale, and the measure is likely to have low precision and reliability when only a few items are included per domain. Fortunately, the advent of computer adaptive testing offers a solution to the ‘time burden’ problem [53, 54].

The unmet need for PROMs in inflammatory eye disease is problematic. The recent SARS-coronavirus-19 global pandemic has ushered in a period of accelerated service transformation in the National Health Service and health systems internationally. This is driving major shifts towards virtual review and remote monitoring and in this context, PROMs could have an important role to play. PROMs improve patient satisfaction with care, symptom management, quality of life and survival rates [55]. The integration of PROM data through technological infrastructure has progressed rapidly leading to the incorporation of internet-based applications, touchscreen tablets and electronic health records into clinical care [56]. For clinicians, PROM collection has been shown to enhance shared decision making by allowing the clinicians to better understand the patient’s symptoms and impact on their quality of life. Furthermore, it can enhance workflow efficiency and save time when used regularly, e.g. by using the limited clinic time to explore a particular symptom burden highlighted from the instrument [57].

### Strengths and limitations

Strengths of this systematic review include adhering to sound systematic review methodology including a comprehensive search for published PROMs and robust quality appraisal of identified instruments. However, we did not extensively search the grey literature or conference abstracts. This means that we might have overlooked reports of unpublished PROMs under current development. Our assessment is that it would be very unlikely that extending to these less developed tools and grey literature would have resulted in the identification and inclusion of any high quality, complete PROMs not identified through the main search. Also, we did not conduct a separate search for all of the immune-mediated inflammatory diseases with which uveitis and scleritis may be associated, or an explicit search for symptom measures. Sets of relevant questions for uveitis or scleritis contained within PROMs designed for associated systemic diseases (e.g. the KSQ), or limited to symptoms, may have been overlooked.

Limitations of the quality criteria we used (eTable 1) were that they held studies that used more modern IRT approaches with PCA to a higher level of account in the grading scheme, than studies which used older and more simple classic test theory approaches. Also, they did not emphasise the relative level of importance of the criteria to one another. Furthermore, the quality criteria did not require assessment of whether or not the patient samples used to develop and to validate a PROM were independent, which is important, so we recommend the inclusion of this as an additional item.

### Implications

The potential value of using a PROM with strong psychometric performance as a trial endpoint cannot be understated. Not only do these permit alignment with the outcomes that most matter to patients, but there are major resource implications. Narrow standard errors around an outcome measure permit recruitment of smaller samples, with major cost saving for trial funders. We identified few PROMs, most of which were developed many years ago and without the benefit of contemporary psychometric approaches. The King’s Sarcoidosis Questionnaire was a notable exception, with the 17-item unidimensional Eye-General Health Status module appearing promising for trials in ocular sarcoidosis, although validation in an independent sample would first be recommended. Based on our quality appraisal, we are not able to recommend any of the currently available PROMs for therapeutic trials in uveitis, or scleritis.

### Future research

Further research to develop robust PROMs for inflammatory eye disease is needed. This would help to address priorities articulated through patient and stakeholder research priority setting initiatives internationally. Namely, to identify, through robust outcome measurement, more effective therapies for rare diseases, including inflammatory eye disease. It will be important for future PROMs to adhere to guidance from the FDA on PROM development [58]. Larger samples of patients are generally needed for content identification and instrument development than have been used in the uveitis PROMs reported here, and these patients should be representative of the clinical phenotypes eligible for the trial. Future studies must ensure independence of development and validation samples, and recruit a sufficient sample size (> 7 × patients than number of items in largest unidimensional scale) for robust psychometric development using the item response theory approach. Investigators may also find the PROTEUS, SPIRIT-PRO and CONSORT-PRO guidelines on the selection and reporting of PROMs for clinical trials helpful [58–61].

## Conclusion

The challenge of developing PROMs, and the dearth of their availability for rare disease areas is well recognised, and applicable to an estimated 5000 to 8000 distinct rare diseases [55, 62]. This systematic review highlights an important, unmet need for the development and validation of PROMs that are able to measure the impact of uveitis or scleritis, and their treatment, on multiple domains of quality of life. Demand for robust PROMs in inflammatory eye disease is anticipated to rise as not only patients and clinicians [57], but regulators, payers, accreditors, and professional organisations recognise their potential value [56]. Given the time and cost taken to develop a new PROM, and the increasingly important role for PROMs both in clinical trials and the modern health service, further research is needed to identify novel ways to reduce the multiple barriers to their development and wider generalisability. This will be essential to capture the outcomes that really matter to people living with these diseases.

## Supplementary Information

**Additional file 1: ePanel 1.** Search strategy for MEDLINE.

**Additional file 2: eTable 1.** Quality appraisal framework for included studies. **eTable 2.** Table providing justification of the assigned quality appraisal gradings.

## Data Availability

Not applicable.
