# Supplementary material for: Evaluating patient-reported outcome measures (PROMs) for clinical trials and clinical practice in adult patients with uveitis or scleritis: a systematic review
Source: J Ophthalmic Inflamm Infect. 2022 Sep 5;12:29. doi: 10.1186/s12348-022-00304-3 (PMC9443634; doi:10.1186/s12348-022-00304-3)
Supplement: Supplementary file 1 — Additional file 1: ePanel 1. Search strategy for MEDLINE. [file 12348_2022_304_MOESM1_ESM.pdf]

Online supplementary files

ePanel 1 Search strategy for MEDLINE

Database: Ovid MEDLINE(R) <1946 to 5 November 2021>

1. quality of life/
2. health status/
3. health status indicators/
4. quality of life.tw.
5. health status.tw.
6. (hql or hqol or hrql or hrqol or qol).tw.
7. (symptom$ adj4 score$).tw.
8. (pros or pro or proms or patient-reported or health related or patient engage$ or patient satisfaction or PROM or patient reported).ti,ab.
9. or/1-8
10. exp Uveitis/
11. uveitis.ti,ab.
12. (inflam* adj3 sclera).ti,ab.
13. exp Scleritis/
14. scleritis.ti,ab.
15. exp Optic Neuritis/
16. optic neuritis.ti,ab.
17. or/10-16
18. 9 and 17
