# Supplementary material for: Evaluating patient-reported outcome measures (PROMs) for clinical trials and clinical practice in adult patients with uveitis or scleritis: a systematic review
Source: J Ophthalmic Inflamm Infect. 2022 Sep 5;12:29. doi: 10.1186/s12348-022-00304-3 (PMC9443634; doi:10.1186/s12348-022-00304-3)
Supplement: Supplementary file 2 — Additional file 2: eTable 1. Quality appraisal framework for included studies. eTable 2. Table providing justification of the assigned quality appraisal gradings. [file 12348_2022_304_MOESM2_ESM.pdf]

eTable 2: Table providing justification of the assigned quality appraisal gradings

Instrument and first author		
**BCR, Barry et al 2014**		
Property	**Grade**	**Explanation for grade**
Item relevance	Very relevant	Birdshot chorioretinopathy is specific cause of posterior uveitis associated with HLAA29 positivity
Item identification	B	"Item development was via face-to-face meetings of the Item Generation Group (composed of 2 patients, consultant ophthalmologist, and a psychologist)"
Item selection	C	Instrument development used factor analysis to identify subscales from the responses of eight patient volunteers and one normal control.
Acceptability	B	"152 responded' ' 'after incomplete responses were eliminated, there were 141 BCR participants"
Item targeting	NR	
Internal consistency	A	"The thresholds for acceptability were: KaiserMeyer-Olkin > 0.6; Cronbach's a > 0.7, and the factor loading threshold of >0.6.4," "For all 3 questionnaires, the thresholds for acceptability for Kaiser-Meyer-Olkin, Cronbach's a, and factor loadings were met or exceeded."
Response Categories	NR	
Dimensionality	NR	
Measurement precision	NR	
Itemfit statistics	NR	
Differential item functioning	NR	
Targeting	NR	
Concurrent validity	NR	
Known group validity	A	"Assessed by comparing the BCR group and control group using Wilcoxon's tests. shows that the BCR group scored significantly lower than controls on the BD&MSQ and QoL BCR scales and subscales, at a minimum P < 0.01. The profile of non-BCR medication was similar in both groups"
Convergent validity	A	"Concurrent validity was also assessed. For the BCR participants, there was a strong negative correlation between the Visual Function Questionnaire Utility Index health state classification and each of the new measures at a minimum of r = -0.546, P < 0.000001, indicating acceptable concurrent validity."
Discriminant validity	NR	
Test-retest repeatability	NR	
Responsiveness	NR	
Independent development and validation?		
**CMV retinitis, Wu et al**		
Property	**Grade**	
Item relevance	Very relevant	Cytomegalovirus retinitis in the context of acquired immunodeficiency syndrome is a specific cause of posterior (or pan) uveitis
Item identification	C	Most items were repurposed from instruments developed for other patient groups, including the Visual Function-14 (VF-14) instrument, developed to assess visual function and symptoms in patients with cataracts; the Medical Outcomes Study Short Form; and the SF-36. Qualitative research was conducted in 18 patients with CMV to identify a few additional items for the 44-item pilot study.
Item selection	B	44-item pilot instrument administered to 26 CMV patients
Acceptability	NR	
Item targeting	NR	
Internal consistency	A	"The new scales had high internal consistency (Cronbach 0.81 to 0.94)"

Response Categories	NR	
Dimensionality	NR	
Measurement precision	NR	
Itemfit statistics	NR	
Differential item functioning	NR	
Targeting	NR	
Concurrent validity	A	"Global vision score was moderately correlated with both visual acuity and visual field (r=0.48-0.63)' 'By means of multiple linear regression, visual symptoms and visual function were independently related to global vision score, each explaining a similar amount of variability in patient reports (total R2=0.78)."
Known group validity	NR	
Convergent validity	NR	
Discriminant validity	NR	
Test-retest repeatability	NR	
Responsiveness	NR	
Independent development and validation?	No	However, subsequent independent validation was reported by Martin et al.
**CMV retinitis, Martin et al**		
Property	**Grade**	
Item relevance	Very relevant	Cytomegalovirus retinitis in the context of acquired immunodeficiency syndrome is a specific cause of posterior (or pan) uveitis
Item identification		
Item selection		
Acceptability	NR	
Item targeting	C	"Few patients reported the lowest possible score (floor) on any of the scales, with the exception of role functioning (61.2%)"
Internal consistency	A	"Cronbach's alpha ranged from from .76 to .87 for the CMV retinitis-specific scales."
Response Categories	NR	
Measurement precision	NR	
Dimensionality	NR	
Itemfit statistics	NR	
Differential item functioning	NR	
Targeting	NR	
Concurrent validity	A	"Vision scores showed a consistent pattern of moderate correlation (r = 0.30–.060) with findings from visual testing and examination (visual acuity, visual field, and extent of involvement)."
Known group validity	NR	
Convergent validity	A	"Each scale's Cronbach's alpha coefficient exceeded its correlation with other scale scores and most items correlated more highly with their own scale than with other scale scores (data not shown)."
Discriminant validity	NR	
Test-retest repeatability	NR	
Responsiveness	A	"Scales of visual function and visual symptoms were responsive to changes over time in findings from visual testing and examination in the better eye (VA in better eye p<0.001 and worse eye p 0.183)"

Independent development and validation?		
**NEI VFQ-25, Mangione et al 2001**		
Property	**Grade**	
Item relevance	**Somewhat relevant**	
Item identification	NR	
Item selection	B	"51-itemNEI VFQ completed by two seperate samples (1st consisting of 262 persons from 5 academic centers who participated in the 1994 pilot test of the NEIVFQ (pilotstudy), and the second sample consisted of 597 persons from the 1996 NEI VFQ Psychometric Field Test"
Acceptability	B	Missing data for each item ranged from 0 to 37%
Item targeting	C	Ceiling effect was >40% in several scores
Internal consistency	A	"Internal consistency estimates for the NEI VFQ-25 subscales ranged from 0.71 to 0.85"
Response Categories	NR	
Dimensionality	NR	
Measurement precision	NR	
Itemfit statistics	NR	
Differential item functioning	NR	
Targeting	NR	
Concurrent validity	A	"Correlations between responses on the NEI VFQ-25 and ETDRS visual acuity were in the range of 0.65 to 0.70 for subscales that reflected degree of difficulty with visual activities related to general vision, near vision, and distance vision'"
Known group validity	A	"152 responded' ' 'after incomplete responses were eliminated, there were 141 BCR participants"
Convergent validity	B	
Discriminant validity	C	"For all 3 questionnaires, the thresholds for acceptability for Kaiser-Meyer-Olkin, Cronbach's a, and factor loadings were met or exceeded."
Test-retest repeatability	NR	
Responsiveness	A	"Visual field loss scores had moderate statistically significant correlations with the NEI VFQ-25 composite score, general vision, distance vision, near vision, peripheral vision, social functioning, dependency, and mental health subscales."
Independent development and validation?		
**NEI VFQ-25 Naik et al 2011/2013a**		
Property	**Grade**	
Item relevance	**Somewhat relevant**	
Item identification	NR	
Item selection	NR	
Acceptability	NR	
Item targeting	NR	
Internal consistency	A	"The NEI VFQ-25 and the VFQ-UI demonstrated good internal consistency (Cronbach's alpha 0.87–0.94)"
Response Categories	NR	
Dimensionality	NR	
Measurement precision	NR	

Itemfit statistics	NR	
Differential item functioning	NR	
Targeting	NR	
Concurrent validity	C	"The correlations for the NEI VFQ-25 with clinical measures of visual acuity (BCVA and vitreous haze) showed The VFQ-UI was significantly correlated with BCVA at baseline, week 8, week 16, and week 26/early exit (r = 0.32, 0.39, 0.37, and 0.36 respectively; all p<0.001), but was not significantly associated with vitreous haze, except at week 26 (r = -0.16, p<0.05)."
Known group validity	A	"Significant pairwise comparisons demonstrated the VFQ-UI was able to detect significant differences between the best visual acuity group (C20/40) compared with those with moderate visual acuity (\20/40–[20/200) and poor visual acuity (B20/200).Significant differences between severity groups in the general vision color vision, and role difficulties domains were observed at week 2"
Convergent validity	C	"All correlations between the NEI VFQ-25 domain scores and the SF-36 domain scores were small to moderate (r = 0.14–0.56)"
Discriminant validity	NR	
Test-retest repeatability	NR	'The ICCs for the VFQ-25 domains ranged from 0.58 (general vision) to 0.83 (near vision and distance vision). Except for the general vision item, the ICCs for all domain scores exceeded 0.70'
Responsiveness	A	"There were statistically significant differences in mean change scores by BCVA change group from baseline to week 8 for the VFQ-UI, NEI VFQ-25 composite, and all NEI VFQ-25 domains, except for general health and driving (all p<0.05)"
Independent development and validation?		
**NEI VFQ-25, Naik et al 2013b**		
Property	**Grade**	
Item relevance	**B**	
Item identification	NR	
Item selection	NR	
Acceptability	NR	
Item targeting	NR	
Internal consistency	A	''The NEI VFQ-25 and the VFQ-UI demonstrated good internal consistency (Cronbach's alpha 0.87–0.94)'
Response Categories	NR	
Dimensionality	NR	
Measurement precision	NR	
Itemfit statistics	NR	
Differential item functioning	NR	
Targeting	NR	
Concurrent validity	NR	
Known group validity	A	"Significant differences between the HURON uveitis population and the normal-vision population were found across all 11 NEI VFQ-25 domain and composite scores and exceeded 10 points (all P <.001)."
Convergent validity	A	"Significant differences between the HURON uveitis population and the normal-vision population were found across all 11 NEI VFQ-25 domain and composite scores and exceeded 10 points (all P <.001)."
Discriminant validity	NR	
Test-retest repeatability	NR	
Responsiveness	NR	
Independent development and validation?		
**KSQ, Patel 2012**		

Property	**Grade**	
Item relevance	Very relevant	Ocular sarcoidosis is associated with dry eye, with anterior, intermediate, posterior and panuveitis, and less commonly, with scleritis and optic neuritis and may cause vision impairment
Item identification	B	Qualitative research on 7 patients with ocular sarcoidosis, plus 16 patients with sarcoidosis affecting other organs, plus literature review, and expert multidiscpliniary opinion. Very unlikely to reach content saturation with so few ocular sarcoidosis patients, and these are unlikely to represent the full spectrum of disease.
Item selection	B	65 item preliminary instrument (including 8 ocular items) administered to 207 patients (including 45 with ocular disease). 45 patients/8 items = 5.6 indicating moderate sample for robust instrument development. Rasch analysis used to develop instrument and identify items with floor effects, high inter-item correlation, disordered response scales, poorly fitting items or differential item functioning with good level of detail reported in main paper and online supplement to justify development of final 29-item instrument.
Acceptability	A	"Ninety-seven percent of patients had no difficulty in completing the KSQ. The mean (SD) time patients took to complete the KSQ was 10 (8) min."
Item targeting	A	Floor and ceiling effects were <5% for Eye-GHS score (Online Supplement Figure 10)
Internal consistency	A	Cronbach's alpha coefficient for eye module +general health status (17 items) = 0.92
Response Categories	A	"The response scale thresholds were re-ordered fo the following items: 1, 3, 5–11, 16–29. The KSQ scores presented in this study were determined using the re-ordered thresholds."
Dimensionality	NR	"The... overall health status scales (combined modules) remained unidimensional following Rasch analysis without eliminating further items: Eye health status (E+GHS)." "The addition of the medications module to the overall Eye health status scale did not fit the Rasch model and the medication module was therefore scored separately in patients with eye disease." Principal components analysis not specifically reported.
Measurement precision	A	Person separation index the eye-GHS was 0.91. We assume this was the reliability coefficient
Item fit statistics	NR	
Differential item functioning	NR	"There was no influence of age, gender, ethnicity, immunosuppressant medication or organ involvement on the response to items (DIF)." The statistics for DIF were not reported specifically.
Targeting	A	The difference between mean item and mean person logits was <1 logit for the Eye-GHS 17 items (Online Supplement Figure 10)
Concurrent validity	C	Tested against appropriate clinical measure (visual acuity) but correlated -0.45. "Multivariable analysis was not performed for Skin, Eye and Medication modules due to insufficient sample size."
Known group validity	NR	
Convergent validity	A	Tested against appropriate clinical measure and correlates 0.8 with NEI VFQ-25, 0.60 with SF36 Physical Component Summary
Discriminant validity	A	Tested against the FAS fatigue PROM and r = -0.82
Test-retest repeatability	A	Investigated in 39 patients (15 with ocular involvement) 2 weeks apart, and intraclass correlation coefficient was 0.96 for the Eye Module (n=7 items). "Bland-Altman plots for the other KSQ modules were also consistent, with the KSQ being a highly repeatable measure."
Responsiveness	NR	
Independent development and validation samples	NR	
